# Cervical Polypectomy Using a Flexible Ureterorenoscope to Preserve the Hymen: A Case Report

**DOI:** 10.7759/cureus.92713

**Published:** 2025-09-19

**Authors:** Wael Elbanna, Asmaa Khedr, Osama Azmy

**Affiliations:** 1 Obstetrics and Gynecology, Hayat Women Care Center, Cairo, EGY; 2 Obstetrics and Gynecology, Egypt Center for Research and Regenerative Medicine (ECRRM), Cairo, EGY

**Keywords:** case report, cervical polyp, flexible single-use ureterorenoscope, hymenal preservation, virgin

## Abstract

Hymenal preservation during the removal of uterine or cervical lesions is crucial for many patients. Techniques like rigid hysteroscopy are effective but cause significant postoperative pain and have limited vaginal access. New procedures are needed to provide effective treatment with less pain, hymenal preservation, and improved patient experience. In this case report, we aim to present a virgin case who underwent flexible laser-equipped single-use ureterorenoscopic cervical polypectomy with preservation of the hymen. A 30-year-old virgin case with a large cervical polyp presented with a history of heavy menstrual bleeding for the past six months, causing severe anemia that required blood transfusion. Her menstrual flow was regular before the last six months. Abdominal examination was unremarkable, whereas pelvic examination showed a polyp protruding through the hymen, which was intact. Transabdominal sonography revealed a cervical mass measuring 50 × 19.7 mm in craniocaudal and transverse diameters, respectively. The magnetic resonance imaging (MRI) findings supported the diagnosis of a cervical polyp. The polyp was successfully removed using flexible single-use ureterorenoscopy with laser energy. This resulted in relief of symptoms and patient satisfaction in terms of adherence to traditions and cultural beliefs. Flexible single-use ureterorenoscopy can be used as an effective alternative to hysteroscopy for giant cervical polypectomy with hymenal preservation in virgin cases. This technique can be considered a therapeutic option for patients wishing to preserve virginity, especially in conservative societies such as the Middle East.

## Introduction

Cervical polyps are common and mostly benign lesions presenting with abnormal vaginal bleeding. They account for 4%-10% of cervical lesions. While they typically occur in multiparous women, they can also develop in nulliparous women [1]. These lesions are usually solid and range in size from a few millimeters up to several centimeters, with the majority being below 2 cm [2]. In rare cases, the polyp size can reach more than 4 cm in the vagina or protrude outside the hymen [3,4]. While there are various management options for cervical polyps, such as dilatation and curettage, electrosurgery, or hysteroscopy, the most commonly used method is hysteroscopy [5].

Hysteroscopy provides accurate, safe, and easy removal of polyps in the outpatient setting. Unlike other treatment options, hysteroscopy offers direct visualization of the uterine cavity under local anesthesia. Different types of hysteroscopes include rigid and flexible hysteroscopes. While the rigid hysteroscope is more widely used, it is associated with more discomfort and pain due to the traumatic passage of its rigid tip through the cervical canal. The flexible hysteroscope provides a less traumatic and painful technique to remove uterine lesions [6].

Hymenal preservation is an important cultural and religious decision for many patients. The gynecologist should fully respect the patient’s will while diagnosing and managing the case. Although the rigid hysteroscope can be used with hymenal preservation, there are still some limitations regarding its use. Given the anatomical positioning of the anteverted uterus, the cervix does not align with the hymenal orifice, making it more challenging to visualize and time-consuming to use a rigid hysteroscope [7]. Thus, there is still a need to find alternative procedures that provide satisfactory surgical outcomes with better patient experience and good visualization. In this report, we aim to present a case of successful polypectomy using a flexible ureterorenoscope in a virgin case without disrupting the hymenal integrity.

## Case presentation

A 30-year-old virgin case presented with a history of heavy menstrual bleeding for the past six months. She had a past medical history of anemia, possibly due to heavy menstrual bleeding. Her past surgical history was limited to tonsillectomy. Her menarche started at the age of 13 years, which was initially regular, and began to become heavy in the past six months.

Progesterone pills were prescribed one month earlier at another clinic to control her bleeding and relieve symptoms after a normal examination showing the polyp protruding from the vagina. However, her symptoms did not improve, and surgical intervention was necessary to remove such a large polyp. The patient was offered surgical options for a cure, which she declined due to concerns about hymenal disruption. The patient and her family refused any intervention that could potentially compromise hymenal integrity. Following that, the patient presented to our clinic seeking further medical advice for curative options with hymenal preservation.

**Figure 1:**
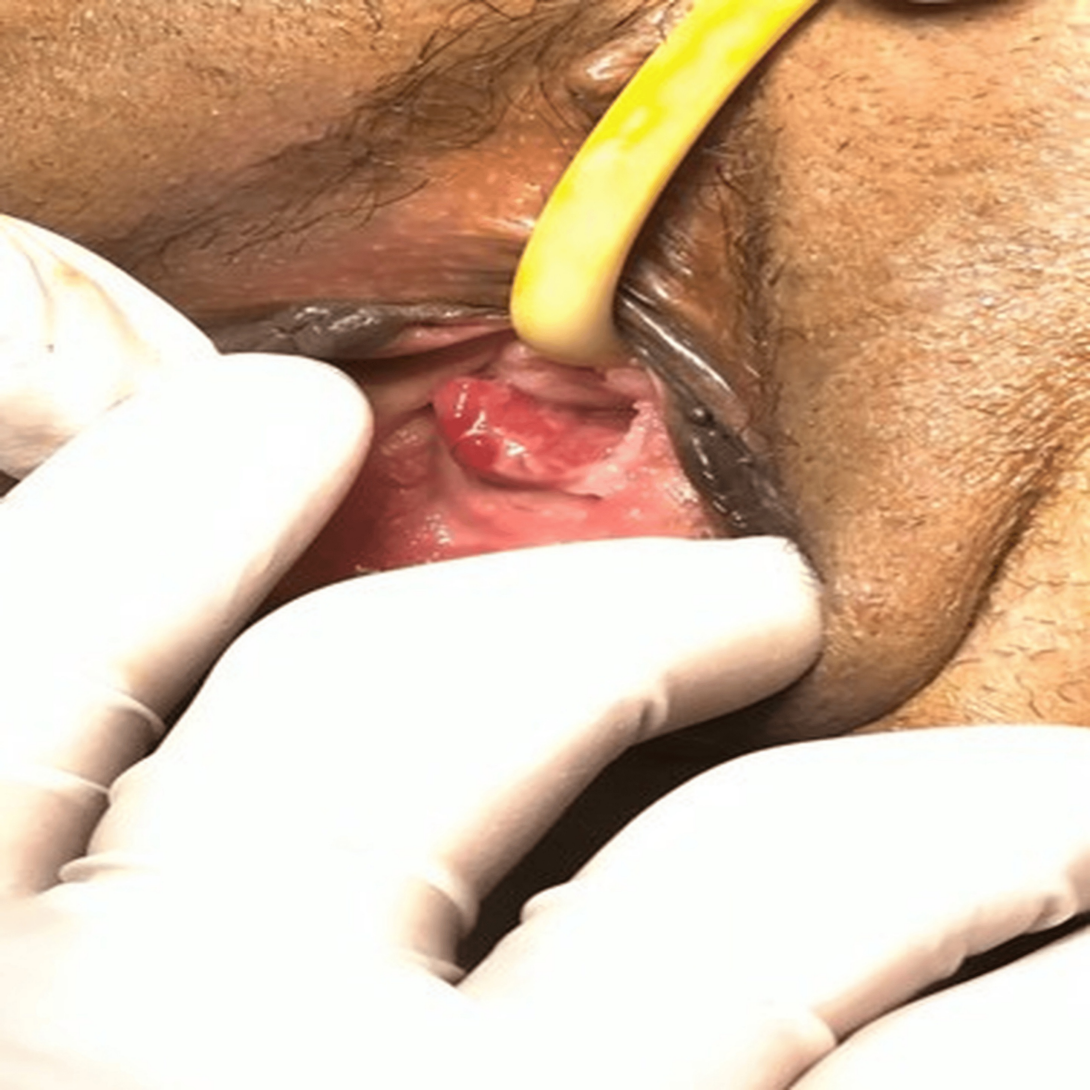
External examination showing a large polyp protruding through the intact hymen

Upon examination, the patient was pale but hemodynamically stable, with blood pressure of 122/74 mmHg, pulse rate of 99 beats/min, and body temperature of 36.9°C. The patient’s weight, height, and body mass index were 71 kg, 163 cm, and 26.72 kg/m². Abdominal examination was unremarkable, and pelvic examination showed a polyp protruding through the intact hymen (Figure 1).

**Figure 2:**
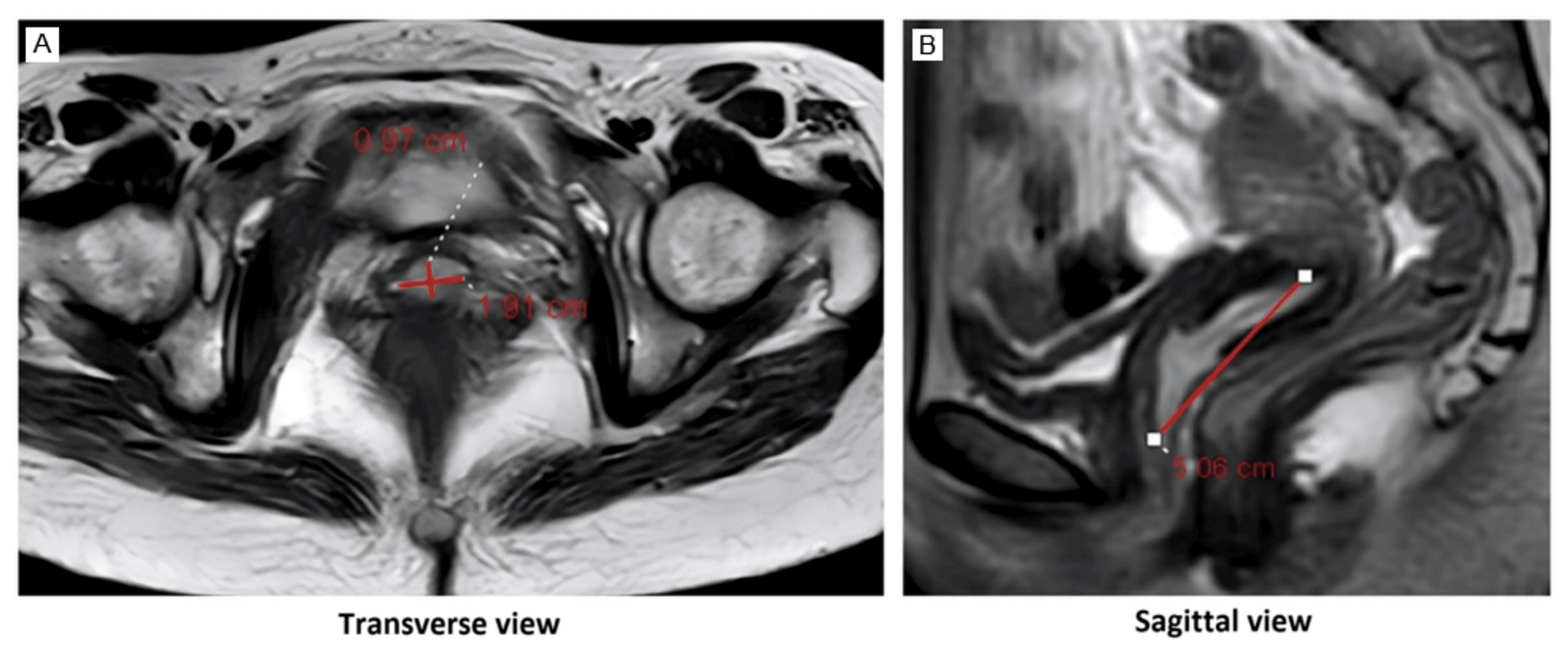
(A, B) MRI scan with contrast showing a large cervical pedunculated cervicovaginal polyp arising from the anterior cervical wall seen widening the cervical canal and extruding from the external orifice to the proximal upper vagina The polyp measures about 5.01 × 1.91 × 0.97 cm in CC, TS, and AP diameters, respectively MRI: magnetic resonance imaging; CC: craniocaudal; TS: transverse; AP: anteroposterior

Transabdominal sonography revealed a normal uterus with a hyperechoic structure occupying the cervical canal measuring 50 × 19.7 mm in craniocaudal (CC) and transverse (TS) diameters, respectively, with free adnexa bilaterally. Magnetic resonance imaging (MRI) showed a large pedunculated cervicovaginal polyp arising from the anterior cervical wall and extruding from the external orifice to the vagina (Figure 2).

These findings were consistent with the diagnosis of a cervical polyp and were explained to the patient. We discussed all available interventional procedures, risks, benefits, and alternatives with the patient and her family. She eventually consented to undergo a flexible single-use ureterorenoscopic cervical polypectomy with laser energy. The patient was informed that the best efforts would be undertaken to preserve the hymen. She was also informed of all potential risks associated with the procedure.

**Figure 3:**
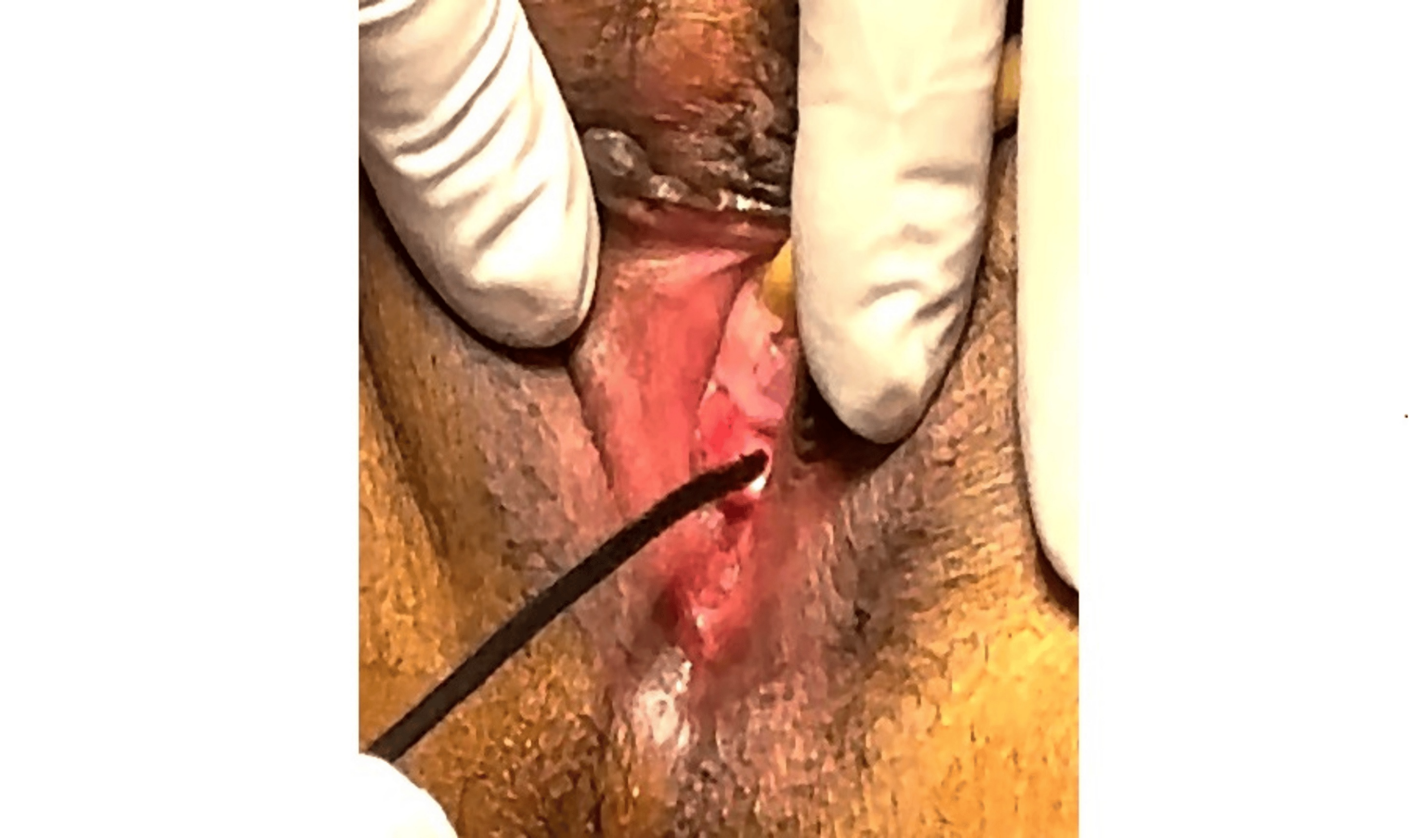
Flexible ureterorenoscopic insertion through the hymenal orifice

Upon preoperative assessment, the patient was in good general condition with stable vital signs. Preoperative investigations were done according to the guidelines [8] and revealed normal parameters. Her preoperative hemoglobin level was 11 g/dL. Under general anesthesia, a 2.5 mm single-use flexible laser-equipped ureterorenoscopy (HugeMed, Single-use Ureterorenoscope, HU30S, US) was inserted into the vagina without a speculum to preserve hymenal integrity (Figure 3).

**Figure 4:**
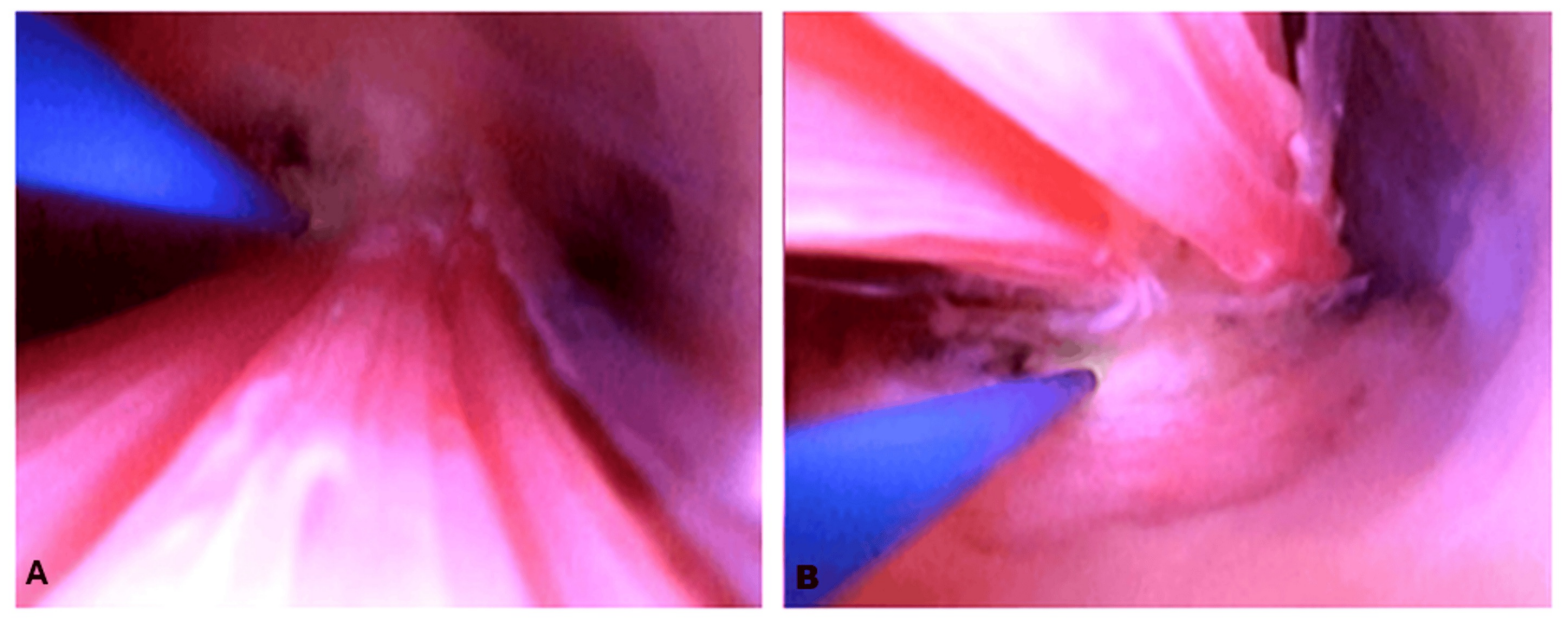
(A, B) Cauterization of the pedicle using flexible single-use ureterorenoscopy with laser energy

Normal saline was used as a distending medium. A polyp arising from the cervix and protruding into the vagina was observed. The cervical polyp pedicle was cauterized using a flexible-fiber holmium-YAG laser with a diameter of 272 μm (Figure 4).

**Figure 5:**
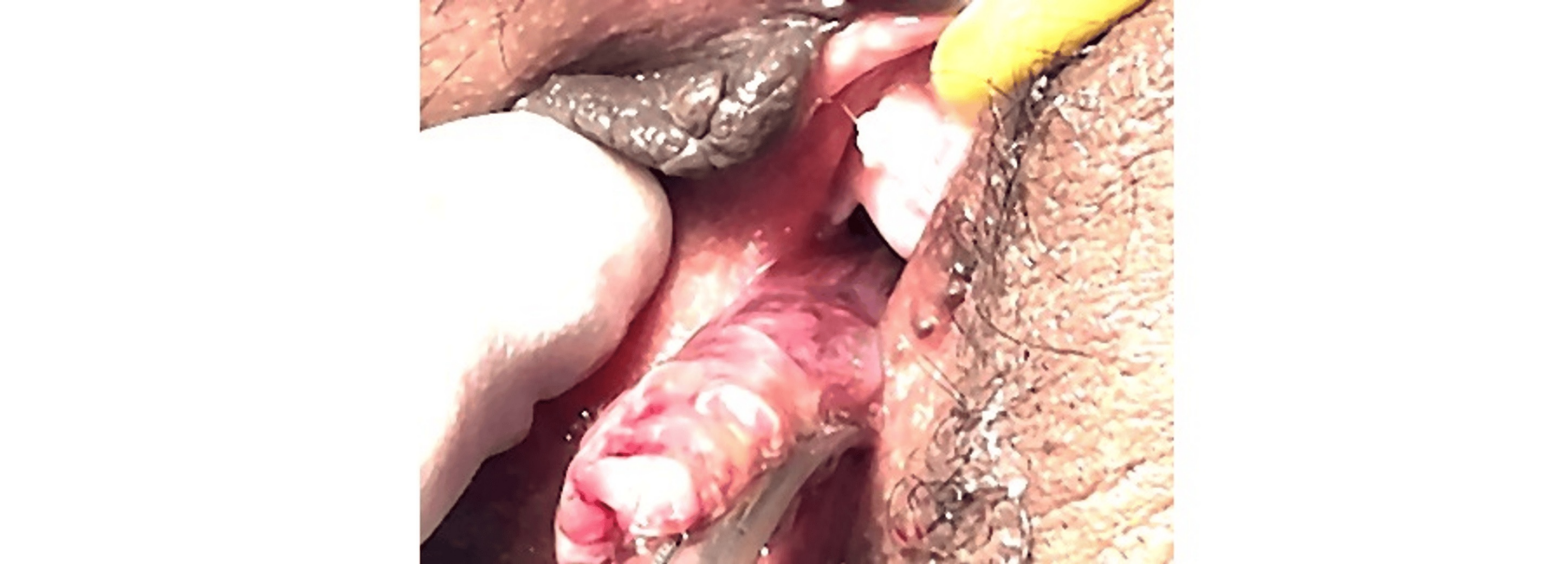
Using polyp forceps through the intact hymen to remove the polyp

The laser settings were of a frequency of 10 Hz and a power of 1-1.2 J, corresponding to 12 W, with a long pulse width of up to 1,500 μs, which was used for cutting through the pedicle of the polyp. Polyp forceps were used through the intact hymen to remove the polyp (Figure 5).

**Figure 6:**
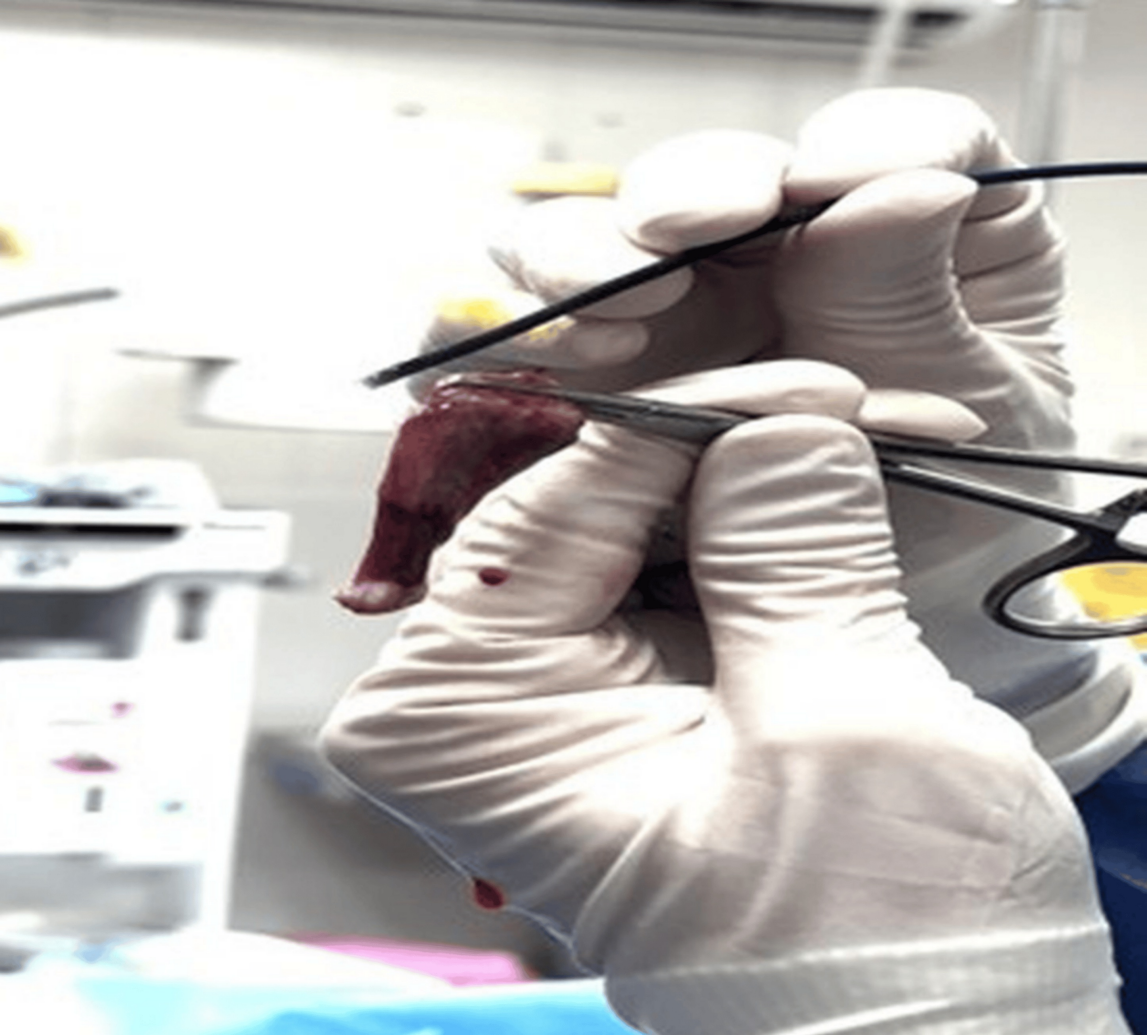
Complete removal of the cervical polyp showing the size of the polyp after resection

The polyp was successfully and completely removed, with a size of 50 × 20 mm in CC and TS diameters, respectively (Figure 6).

The histopathological examination of the excised cervical polyp revealed a gray-pink, rubbery polypoidal tissue measuring 4.5 × 1 × 0.5 cm. Microscopically, the surface was focally ulcerated, showing partial squamous metaplastic changes. Their cores showed inflamed fibrovascular stroma with dilated mucin-secreting glands. These findings were consistent with an adenofibromatous polyp with mucous retention cysts, focal ulceration, and chronic inflammation.

The integrity of the hymen was verified after the surgical procedure and found to be intact. The procedure took 22 minutes. There was no intraoperative bleeding or need for blood transfusion. Postoperatively, the patient was placed in the recovery room for observation and was found to be in good and stable general condition. There was no postoperative bleeding or need for blood transfusion. Her postoperative pain score, measured by the visual analogue scale (VAS), was 1. The patient received non-steroidal anti-inflammatory drugs for pain management when needed. Her hemoglobin level on the first postoperative day was 10.5 g/dL. The patient was discharged on the same day, 12 hours after the procedure. Upon postoperative follow-up visits, after three and six months, her discomfort sensation subsided, and her menstrual flow returned to normal, confirming resolution of symptoms and no recurrence.

## Discussion

Cervical polyps are relatively common and are usually benign. They can occur in women of all ages, including virgins [9]. The symptoms may include intermenstrual bleeding, postcoital bleeding, or discharge. However, many cervical polyps are asymptomatic and are discovered incidentally during routine gynecological examinations. Diagnostic approaches include non-invasive imaging, as transabdominal ultrasound can be used to visualize the cervix and identify polyps without compromising the hymen [10].

Although rigid hysteroscopy and alternative approaches such as mini-hysteroscopy are considered for cervical polypectomy, they were consistently declined by the patient whose cultural background emphasized the importance of preserving hymenal integrity due to the potential risk of its disruption. The flexible single-use ureterorenoscope is an emerging and increasingly used device in urinary tract procedures due to its high deflection capability and low costs [11]. However, there is no data regarding its use in gynecological procedures. It was selected for its flexibility, which enables hymenal preservation while providing superior visualization and minimizing trauma compared to rigid hysteroscopy. Thus, it could be a promising alternative for virgin cases, especially in conservative societies such as the Middle East, which are against any hymenal intervention before marriage.

In this case, we present an innovative technique using a flexible ureterorenoscope for managing a cervical polyp in a virgin, unmarried case. The polyp caused significant bleeding and, thus, could not be left untreated. Our patient and her family were vehemently against any surgery that may disrupt the hymenal integrity and adamant about preserving her virginity. With proper patient counseling, she decided to proceed with the transhymenal approach. The potential risks, such as bleeding or period-type pelvic pain, and the anticipated benefits, such as less tissue trauma, less pain than rigid hysteroscopes, and hymenal preservation [11,12], were discussed with the patient and her family, and the patient consented to the procedure. We managed to maintain the integrity of the hymen using a 2.5 mm single-use flexible laser-equipped ureterorenoscope (HugeMed) without a speculum.

From the patient's perspective, she was filled with a profound sense of relief after the procedure. Not only had the surgery successfully removed the polyp, but she also expressed feeling empowered that she had maintained her virginity. She shared that this experience reinforced her belief that it is possible to prioritize health while staying true to one’s cultural and religious principles. The ease of performing this procedure may depend on the hymenal type. In patients with cribriform or microperforate hymens, the approach could be more challenging.

The preservation of hymenal integrity and the minimal postoperative vaginal bleeding on follow-up highlight the effectiveness of this technique. Although research on reproductive and sexual health reports that virginity is not an anatomical feature, such language could not be avoided in the manuscript, given the culture the patient comes from.

## Conclusions

Cervical polypectomy using a flexible, single-use ureterorenoscope with laser energy provides a promising alternative to traditional techniques for patients seeking hymenal preservation. This approach showed safety, effectiveness, cultural acceptability, and successful removal of cervical polyps while maintaining hymenal integrity and minimizing postoperative pain.

Further studies are needed to validate this technique and assess its reproducibility. It will guide the establishment of clear guidelines for its use in gynecologic settings where preserving virginity is a critical consideration.
